# What Is the Value of Water Contact Angle on Silicon?

**DOI:** 10.3390/ma13071554

**Published:** 2020-03-27

**Authors:** Paweł Bryk, Emil Korczeniewski, Grzegorz S. Szymański, Piotr Kowalczyk, Konrad Terpiłowski, Artur P. Terzyk

**Affiliations:** 1 Department of Chemistry, Chair of Theoretical Chemistry, Maria Curie-Skłodowska University, 20-031 Lublin, Poland; pawel.bryk@gmail.com; 2 Faculty of Chemistry, Physicochemistry of Carbon Materials Research Group, Nicolaus Copernicus University in Toruń, Gagarin Street 7, 87-100 Toruń, Poland; e.korczeniewski@umk.pl (E.K.); greg_ss@umk.pl (G.S.S.); 3 College of Science, Health, Engineering and Education, Murdoch University, Murdoch WA 6150, Australia; kowalczyk.piotr@wp.pl; 4 Department of Chemistry, Chair of Physical Chemistry of Interfacial Phenomena, Maria Curie-Skłodowska University, 20-031 Lublin, Poland; terpil@poczta.umcs.lublin.pl

**Keywords:** wetting, silicon, contact angle, molecular dynamics simulation, hydrocarbons hypothesis

## Abstract

Silicon is a widely applied material and the wetting of silicon surface is an important phenomenon. However, contradictions in the literature appear considering the value of the water contact angle (WCA). The purpose of this study is to present a holistic experimental and theoretical approach to the WCA determination. To do this, we checked the chemical composition of the silicon (1,0,0) surface by using the X-ray photoelectron spectroscopy (XPS) method, and next this surface was purified using different cleaning methods. As it was proved that airborne hydrocarbons change a solid wetting properties the WCA values were measured in hydrocarbons atmosphere. Next, molecular dynamics (MD) simulations were performed to determine the mechanism of wetting in this atmosphere and to propose the force field parameters for silica wetting simulation. It is concluded that the best method of surface cleaning is the solvent-reinforced de Gennes method, and the WCA value of silicon covered by SiO_2_ layer is equal to 20.7° (at room temperature). MD simulation results show that the mechanism of pure silicon wetting is similar to that reported for graphene, and the mechanism of silicon covered by SiO_2_ layer wetting is similar to this observed recently for a MOF.

## 1. Introduction

The process of cleaning is a crucial stage in the study of surface properties, and more or less advanced cleaning procedures have been proposed prior to the contact angle (CA) measurements to remove the adsorbed oil, hydrocarbons, and other impurities [1]. It has been well documented experimentally that physical adsorption of hydrocarbons by changing a surface hydrophobicity can simultaneously affect the values of CA especially if water droplet is sitting on a surface. As it was shown by us recently (see, e.g., in [2] and references therein) the influence of airborne adsorbed hydrocarbons on water contact angle (WCA) depends on the coverage and the nature of a surface. Generally, it was shown that the increase or a decrease in the WCA value after airborne hydrocarbons adsorption can be observed. Recently, we also proved that if the affinity of water to a substrate is high water nanodroplet can completely remove light hydrocarbons from a surface and robust self-cleaning properties of a surface are observed [3]. There are few major topics that should be considered during silicon surface wetting. As it was pointed out by de Gennes et al. [1], it is well known that a silicon surface is covered by a thin layer of SiO_2_. This thin oxide layer can be removed [4] by a treatment of Si with HNO_3_, HF, and CH_3_COOH mixture; however, after this process, a sample should be stored in an inert atmosphere. Moreover, the silicon surface is additionally covered by this layer of adsorbed hydrocarbons. Shinozaki et al. [5] studied the methods of SiO_2_ surface cleaning from adsorbed hydrocarbons, using three different methods of surface cleaning. The thickness of adsorbed hydrocarbons layer is in the range of ~0.2 nm. Choi et al. [6] showed that the UV/O_3_ plasma treatment removes adsorbed hydrocarbons completely. However, during a plasma treatment, surface roughness appears and it is more visible for longer plasma treatment time. This roughness influences the CA values. Due to the above-mentioned reasons, WCA reported experimentally and obtained by molecular simulations for silicon differ remarkably. For example, the WCA equal to 86–88° was reported from experimental studies for etched silicon [7]. Computer simulation results of a wetting process of a (0,0,1) SiO_2_ surface [8] led to WCA equal to 24 ± 1.28°. Han et al. determined the WCA on a smooth silicon surface as equal to 54° [9]. Isaev et al. [10] assumed that WCA is in the range of 87°. Similar WCA value on silicon was obtained from simulation by Barisik and Beskok [11]. These authors assumed a Stillinger–Weber potential for silicon and the SPC/E model of water. They quote [7] a range between 86 and 88° as the true experimental WCA on etched silicon. However, as we mentioned above, a fresh silicon surface quickly develops a ~1.4–1.5 nm thick layer of silicon dioxide, leading to significant lowering of WCA. One of the conclusions of the Barisik and Beskok study is that it is important to include the Stillinger–Weber potential between silicon atoms in order to get the correct wetting behavior of this system. The key argument (cf. Figure 4 in [11]) is that WCA as a function of the substrate–water interaction strength (ε) develops two wetting regimes characterized by different slopes of the plot. Unfortunately, we find this argument to be flawed. As it is well known [12,13], there is a linear dependence of cosθ (where θ = CA) vs. ε (and not θ vs. ε) close to the first order wetting transition, i.e., for small values of 1-cosθ [2]. Although this relationship in practice persists up to fairly large WCA values, for systems sufficiently well removed from the wetting transition, the plot of cosθ vs. ε will develop a curvature (see [14]). The inclusion of silicon–silicon interactions will lead to vibrations of the silicon lattice substrate structure and will alter water particles in the nearest vicinity of the surface but this cannot qualitatively alter the wetting properties of the silicon. Adding insult to injury, Barisik and Beskok [11] used spherical droplets; therefore, their WCA values can be affected by the line tension contribution [15]. It is well known that this contribution can have different signs depending on the value of the CA, with a positive diverging value close to the wetting transition [16]. Summing up simulation approaches does not consider the onset of the silicon dioxide thin layer, nor do they consider the presence of the airborne surface hydrocarbons and their influence on CA value. This presence must be taken into account because the CA is usually measured in the air atmosphere. The aim of our study is to present a holistic experimental and theoretical approach to evaluate the effect of alkane adsorption on water–silicon CA. First of all, we present the experimental results. Different silicon surface cleaning methods are applied and, after cleaning, the WCA is measured in an inert as well as in hydrocarbons atmosphere. To confirm the influence of hydrocarbons some chromatographic measurements are additionally performed. Finally, the results of Molecular Dynamics (MD) simulations are reported to determine the wetting mechanism in light hydrocarbons atmosphere and to obtain the correct force field parameters leading to agreement between experiment and simulation.

## 2. Materials and Methods

Three types of silicon samples were used in our experiment: A (1,0,0) plate (ON Semiconductor Co, Raznov, Czech Republic, obtained by using Czochralski method) cut on squares (10 × 10 mm) using a diamond edge; a (1,1,1) silicon wafer (ITME, Warszawa, Poland) obtained by using a Float Zone method and laser cutting (7 × 7 mm); and a silicon powder (purity 5N-99.999%), 100–200 µm, produced by Sabon Plant, Silicio FerroSolar SLU, Madrid, Spain. The (1,0,0) and (1,1,1) samples were applied in the X-ray photoelectron spectroscopy (XPS) and wetting studies, and the powder in the chromatographic study. XPS spectra were measured using the UHV multi-chamber analytical system from Prevac Ltd. (Rogów, Poland). The system has been equipped with sources (MX-650 from Gamma data Scienta, anode Kα-Al) or achromatic X-rays (anode: Mg/Al or Ag, resolution <1 meV). Before WCA measurements, three separate purification processes were performed: the procedure taken form original de Gennes book [1] (labeled as DG), the DG procedure followed by a pre-treatment with solvents (called SDG), and the procedure of purification in acetone (samples were labeled as A and LA, respectively). In the DG method, the plates (stored in polypropylene boxes) were removed from a polyvinylchloride tape and placed immediately in an acidic bath containing H_2_SO_4_/H_2_O_2_ (30 min, 70:30, H_2_SO_4_—95% pure p.a., Chempur, Piekary Śląskie, Poland, H_2_O_2_—30%, POCH, Gliwice, Poland) following the procedure proposed in [1].

During the SDG method, a silicon sample was removed from a polyvinylchloride tape and placed in ultrasonic bath in acetone (pure p.a., Chempur, Piekary Śląskie, Poland) for 45 min (after 30 min. the solvent was removed and replaced by a fresh one). Next, each silicon plate was washed out using the surfactant Rosulfan L (PCC Exol SA, Brzeg Dolny, Poland). This surfactant is composed of sulfuric acid, C12-14-alkyl(even numbered) esters, and sodium salts. Next, the samples were washed out with deionized water (containing 3–6 ppm of ions) and placed immediately in an ultrasonic bath in ethanol (pure p.a., Chempur, Piekary Śląskie, Poland) for 30 min (after 15 min. the solvent was removed and replaced by a fresh one). Next, ethanol was replaced by isopropanol (pure p.a., Chempur, Piekary Śląskie, Poland, 30 min; after 15 min. the solvent was removed and replaced by a fresh one). Finally, the same was done with acetone (pure p.a., Chempur, Piekary Śląskie, Poland, 20 min; after 10 min. the solvent was removed and replaced by a fresh one). Next, the DG procedure standard described above was performed.

Acetone purified materials were obtained by placing a silicon sample in acetone (pure p.a., Chempur, Piekary Śląskie, Poland) for 3 days (sample labeled as A) and for one month (LA).

Finally, the samples from all studied series (excepting A and LA) were washed out at least 5 times in deionized water and placed in small glass containers (each sample separately) in an oven (373 K, 30 min). Each sample was next cooled for 4 min in a closed cell. Next, the initial WCA was measured, and the samples were subjected to the C_10_H_22_ atmosphere following a similar procedure to that described in detail previously [2]. WCA was measured at 298.15 K (at relative humidity equal to 40 %). A goniometer was equipped with a camera Grasshopher3 GS3–U3–32S4C–C, 3.2 Mpx connected to the specially designed optical system consisting of perfectly located in the optical axis two elements: Edmund Optics 1.0x Telecentric Lens 55350 and Edmund Optics Telecentric Illuminator Lens 62760 with a bright diode placed in it (MicroBrite ™ Spot/Coaxial Light model: SL223 470 IC, Light wavelength: 470 nm) leading to polarized parallel light. Between these two optical elements, a precise measuring cuvette made of optical glass (Hellma Analytics, Optical Glass, Light Path 50 mm, model: 704-003-50-10) was placed in the optical axis, having a glass lid with a precise hole with a diameter corresponding to the outer diameter of the dispensing needle. Drops were dispensed using a syringe dispenser manufactured by CERKO, Poland, Gdynia, equipped with a Hamilton syringe, enabling precise dosing of the set volume of the measuring liquid. The needles used were disposable needles manufactured by John P. Kummer GmBH (Needle Tip all in PTFE, size G30, in length 2 inches, model: PDS-Z592130). The probe liquid was deionized water with an ion content of 3–6 ppm, thermostated at the measurement temperature for 30 min. Ten microliter drops were used for all measurements. Liquid hydrocarbon was added to the system in a volume of 1 mL into a small weighing vessel placed inside the cuvette described, with a constant evaporation surface of 260 mm^2^ each time. Between each measuring series, the entire measuring cuvette was thoroughly cleaned using detergents and acetone, and next dried in an ambient atmosphere. Static contact angles were measured based on the obtained images using the ImageJ software with plugin Drop Analysis -LB-ADSA method [17] with optimization of all available parameters through gradient energy approach. Each measurement was repeated at least 3 times using fresh silicon samples and reagents. For the samples from the A and LA series, the WCA was measured immediately after acetone evaporation.

Finally, we also checked the method of purification in HF however, the AFM analysis of studied samples revealed strong roughness and this is why the samples purified using this method were excluded from our study. No roughness creation was observed for the samples purified using other methods.

The chromatographic investigations were carried out using a Chrom 4 gas chromatograph with a flame ionization detector (FID) using helium as a carrier gas. A computer was connected to the gas chromatograph to control, acquire, and process the chromatographic data. The adsorbent was placed in a glass column (50 cm × 2 mm I.D.) with an absorbent bed length of 40 cm, which corresponded to 3.15 g of silicon used (particle diameter of 0.1–0.2 mm). Before the adsorption experiments, the column with the adsorbent was conditioned at 423K for 1 h under a flow of helium. N-decane and n-pentadecane (pure p.a., Chempur, Piekary Śląskie, Poland) were used as adsorbates. The adsorbates were injected into the column by means of a Hamilton microsyringe. The size of the injected samples was 0.2 µL. The temperature of the injection device was set at 423 K and 453 K for n-decane and n-pentadecane, respectively. Additionally, in some experiments, to investigate the effect of water on hydrocarbons adsorption, different amounts of water (1.2 µL) were dosed before a hydrocarbon injection. The reagent injections were performed in the range of 333 to 353 K (n-decane) or 373 to 423 K (n-pentadecane) at carrier gas flow rate 12 cm^3^ min^−1^ (measured by means of a bubble gauge).

To perform theoretical studies, we model the silicon substrate as layers of frozen particles interacting via the Lennard–Jones (12–6) potential with the oxygen from the SPC/E model of water. The silicon atoms of diameter σ_Si_ = 2.095 Å are arranged on a diamond cubic lattice with 5.431 Å spacing. All dispersion interactions have a cut-off radius r_cut_ = 15 Å. We use the SPC/E [18] model of water with σ_OO_ = 3.166 Å, ε_OO_ = 0.6497752 kJ/mole, q_O_ = −0.8476, q_H_ = 0.4238, HOH angle 109.47°, and OH bond length 1 Å. We assume the additivity of diameters σ_ij_ = (σ_ii_ + σ_jj_)/2. However, the dispersion interactions between the silicon and oxygen are weaken, ε_SiO_ = k_d_(ε_SiSi_ε_OO_)^1/2^ with ε_SiSi_ = 209.199544kJ/mole. The parameter k_d_ will be estimated numerically in order to recover the experimental values of WCA. The system of SPC/E water molecules is first arranged on a parallelepiped lattice and placed on the silicon substrate. A minimization of the potential energy is performed followed by molecular dynamics simulation in the NVT ensemble (with time step 0.002 ps) using Nose–Hoover thermostat (τ = 0.1) and SHAKE algorithm for keeping the water molecules rigid. Equilibration was performed for 2 ns while the time averages were accumulated by at least 40ns (up to 60ns for very small WCAs).

Long-range Coulomb interactions were evaluated using the Particle Mesh Ewald (PME) method with 15 Å taken as a value demarcating the real-space and k-space PME calculations. All simulations were carried out using OpenMM molecular simulation package [19,20,21,22,23] at constant temperature T = 298.15 K. The SPC/E water on pure silicon calculations were divided into three phases: First we evaluated the k_d_ parameter using cylindrical droplets, so that the experimental value [7] of the WCA on silicon are recovered in simulation. To this end, we prepared a parallelepiped silicon surface of dimensions 543 Å × 32.58 Å x 16.29 Å (x,y,z dimensions, respectively) containing 15,600 Si atoms with (1,0,0) plane being the top surface facing in the z-direction. On the top of this surface, 3971 SPC/E water molecules were placed. The simulations were carried out at constant temperature T = 298.15 K, and the simulation box was enclosed by a cylindrical repulsive wall of radius 150 Å. From the positions of the water molecules the two dimensional density profiles (Cartesian grid) of the cylindrical droplets were calculated. The WCA of cylindrical droplets was determined by finding the density contour satisfying 0.5 ± 0.03 g/cm^3^.

## 3. Results

### 3.1. Silicon Characterization and WCA Measurements

**Figure 1:**
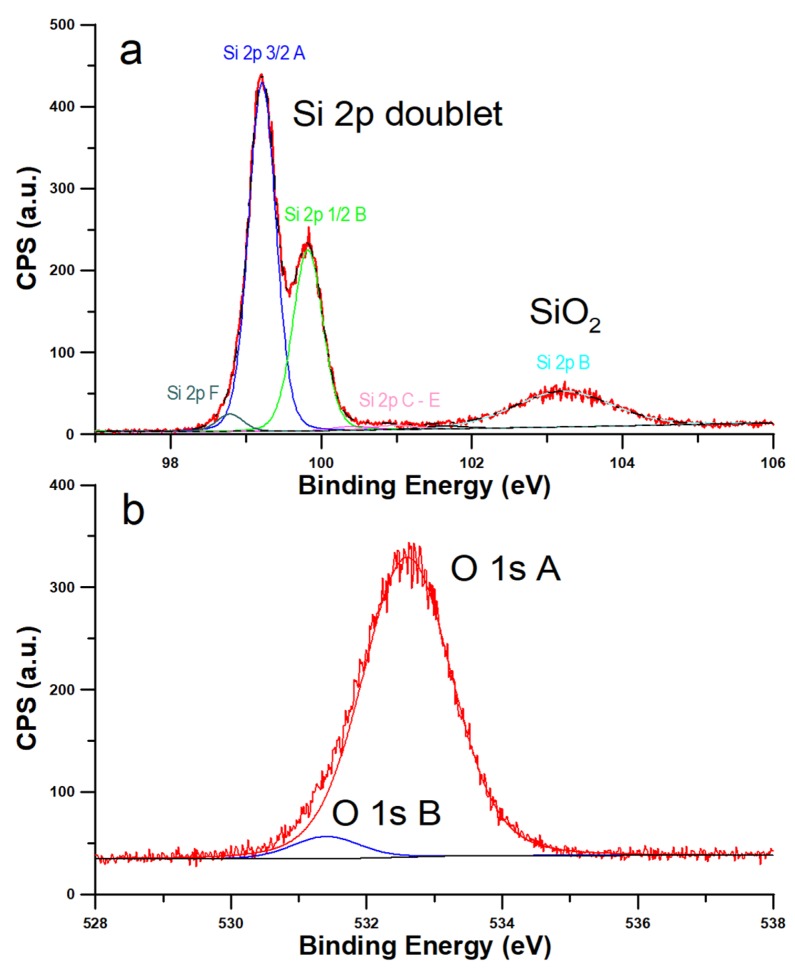
Deconvolution of the Si_2p_ (**a**) and O_1s_ signals (**b**) of the Si (1,0,0) wafer.

The results of the XPS measurements for a typical silicon sample are shown in Figure 1. High-resolution XPS spectra recorded the following elements; carbon, oxygen, sodium, chlorine, and silicon. Elements such as carbon, sodium, and chlorine were found in trace amounts. In the case of silicon, on the surface there is a 78.4 mass concentration of silicon and 16.7 of oxygen. These two components are crucial for experiment [24]. The Si 2p B peak appeared at 103.2 eV and it is associated with the Si (+IV) state corresponding to SiO_2_ structure on the surface (Figure 1, Table 1). Pure Si bonds were detected at 99.22 and 99.82 eV. 24.7% of silica occurs on the surface in combination with oxygen [25,26].

Summing up, it is seen that on the surface of our samples the SiO_2_ layer is created.

**Figure 2:**
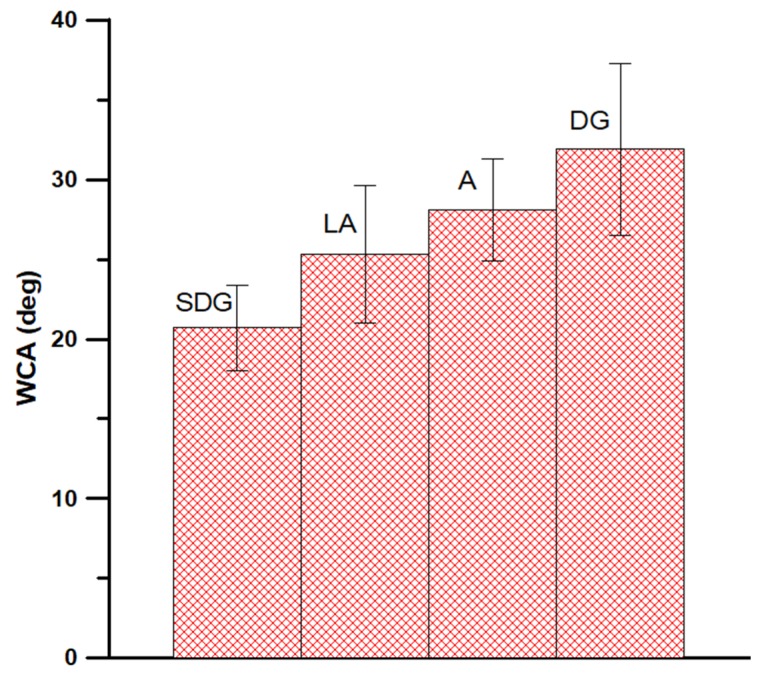
The average water contact angle (WCA) values for silicon (1,0,0) samples subjected to C_10_ H_22_ exposition at 298.15 K.

The results of measurements in C_10_H_22_ atmosphere show no remarkable differences between the (1,0,0) and (1,1,1) samples. Moreover, we do not observe the dependence of WCA on hydrocarbon exposition time, and only oscillations of WCA values around an average value (this behavior will be explained by the results of MD simulations, see below). Figure 2 presents the comparative analysis of the average WCA values measured for (1,0,0) samples purified using different methods. One can observe that the differences in WCA depend on the method of purification, and the smallest value, i.e., 20.7 ± 2.7° is observed for the samples cleaned using the SDG procedure. This value is assumed for the scaling of the force field during MD simulations (see below).

### 3.2. Chromatographic Measurements

**Figure 3:**
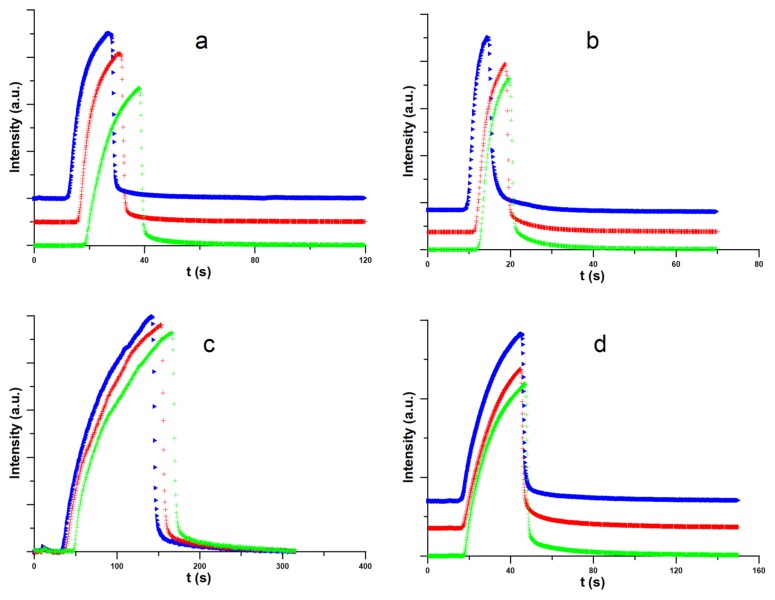
Chromatograms of dosed 0.2 μL n-decane ((**a**) 333 K; (**b**) 353 K) and 0.2 μL pentadecane ((**c**) 373 K, (**d**) 398 K), without (green lines) and after the injection of 1 μL (red line) and 2 μL (blue line) of water.

The aim of the chromatographic results was to confirm that water repels light hydrocarbons from a silicon surface. In fact, the results collected in Figure 3 confirm this hypothesis, as we observe the progressive decrease in both hydrocarbons retention times after injection of water to the system. This phenomenon is a function of temperature and hydrocarbon type, and as it was experimentally evaluated by us that for the system n-pentadecane water it vanishes at ~423 K.

### 3.3. Molecular Simulations of SPC/E Water on Silicon

**Figure 4:**
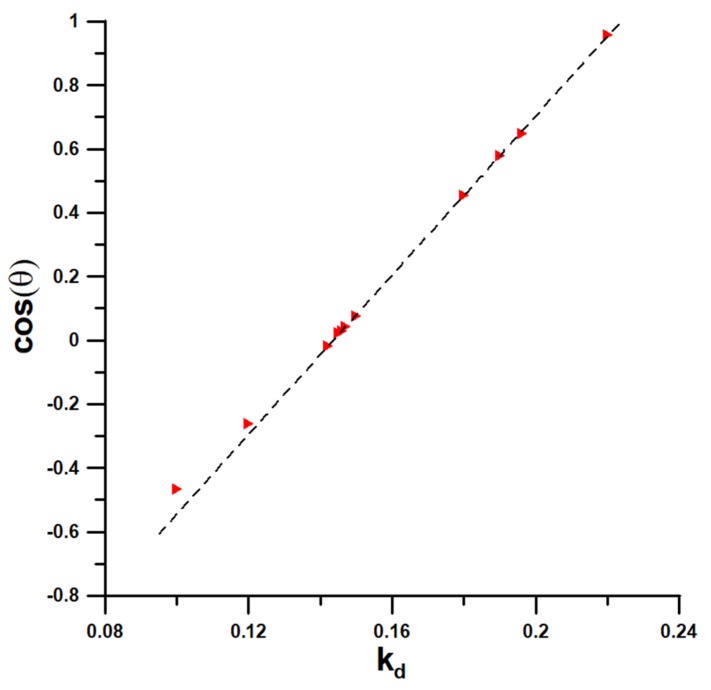
Dependence of the simulated cosine of WCA vs. k_d_ at 298.15 K.

Figure S1 shows a water contour calculated for k_d_ = 0.147. The bottom of the droplet is defined as a part of the density profile near the surface with the density of at least 0.5 g/cm^3^ and from this figure we identify this at z = 17.6 Å. Next, the least-square method of fitting the contour to the circle was applied, taking the points that are at a distance at least 10 Å from the surface. Figure S2 shows the evolution of the WCA with time for k_d_ = 0.147. In general, the variation of WCA is within the bracket of experimentally reported values [7]. The calculations were carried out for a set of different water–silicon interaction strengths: k_d_ = 0.1, 0.12, 0.142, 0.145, 0.146, 0.147, 0.15, 0.18, 0.19, 0.196, and 0.22. We note that the dependence of WCA vs. k_d_ varies from 117.7 for k_d_ = 0.1 (corresponding to ε_SiO_ = 1.1659 kJ/mole) to 16.8 for k_d_ = 0.22 (corresponding to ε_SiO_ = 2.5650 kJ/mole) and it is nonlinear. Instead we find nice linear relationship for the dependence of the cosine of WCA vs. k_d_, see Figure 4.

From this figure we estimate the value of the substrate–water interaction strength at which the first order wetting transition takes place k_w_ = 0.2237 or ε_SiO_,_w_ = 2.6081 kJ/mole. For the largest CA values we find that the deviations from linearity set in, as expected. This figure confirms the quality of the obtained results. The calculations presented above indicate a good silicon–water interaction strength k_d_ = 0.147, or ε_SiO_ = 1.71387548725 kJ/mole. This value has been used in subsequent calculations.

**Figure 5:**
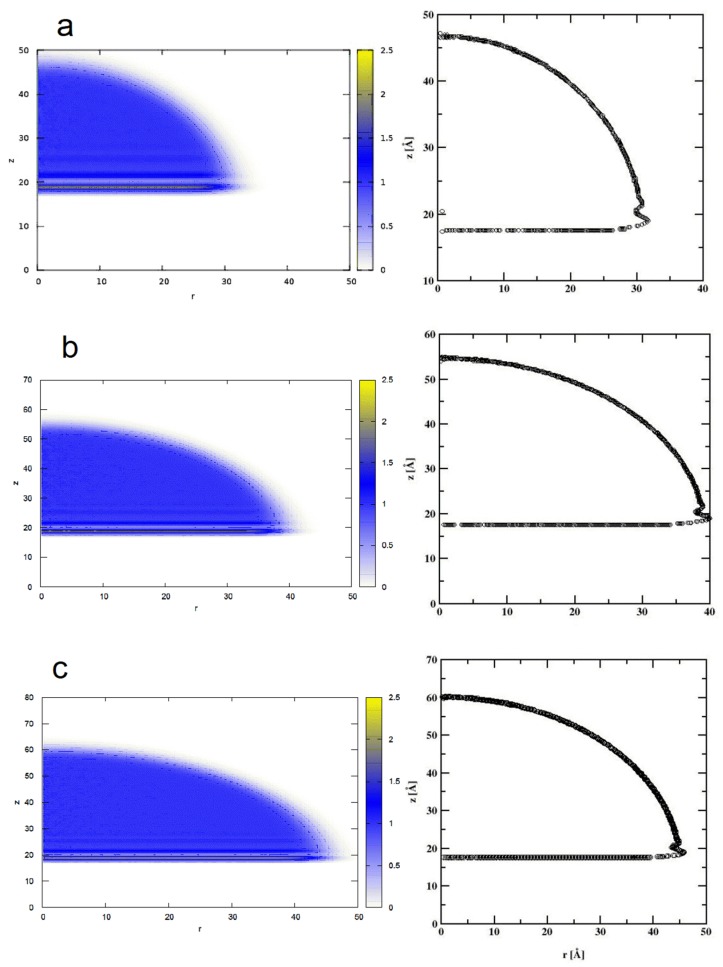
Map of the mass density profiles (left column) and the spherical droplets contours (right column) of water droplet on bare silicon surface, for the droplets consisting of 2000 (**a**), 4000 (**b**), and 6000 (**c**) water molecules. The circles denote the position of water density profile with value 0.5 ± 0.03 g/cm^3^.

In the second stage of calculations, we estimate the influence of the finite system size (i.e., the effect of the line tension on the WCA). To do this we created a surface of Si of dimensions 21.74 nm × 21.74 nm × 1.629 nm (x,y,z dimensions, respectively) containing 41,600 Si atoms with 100 top plane facing the z-direction. On the top of this surface we placed a certain number SPC/E water molecules. Similar to the simulations for cylindrical droplets the simulations of the spherically symmetric drops were also carried out at constant temperature T = 298.15 K with the same thermostat parameters. The simulation box was topped by a spherical repulsive wall of the radius 10 nm. Once the initial minimization of the positions of water molecules was accomplished, the spherically symmetric drop is readily formed allowing the calculation of the radially symmetric density profile. From the radially averaged two-dimensional density profile ρ(r, z), we evaluated the density contours satisfying 0.5 ± 0.03 g/cm^3^. Figure 5 shows the water drop contour calculated for k_d_ = 0.147 and for three different numbers of water molecules, N_SPC/E_ = 2000, 4000, and 6000, respectively.

Similar to the cylindrical droplet, the bottom of the spherical droplet is defined as the part of the density profile near the surface with the density of at least 0.5 g/ cm^3^, and again from Figure 5, we infer it to be z = 17.6 A.

Likewise, the least-square method of fitting the contour to the circle was applied, taking the points that are at a distance at least 10 Å from the surface. We find the WCA values of 86.84°, 86.83° and 86.41° for the spherical droplets comprising of 2000, 4000, and 6000 water molecules, respectively. These results show that there is very little dependence of the WCA on the droplet size. Consequently, the line tension finite size effects are negligible when considering droplets consisting of 6000 water molecules.

In the third phase of calculations, we simulated the water drops on alkane preadsorbed silicon surfaces. The simulation system was prepared by creating a surface of Si of dimensions 21.74 nm × 21.74 nm × 1.629 nm (x,y,z dimensions, respectively) containing 41,600 Si atoms with 100 top plane facing the z-direction, i.e., the same number of silicon atoms as for the spherical water drop-bare silicon surface systems. Next, on the top of silicon surface we placed a number of n-decane molecules in order to achieve the required surface density. We use the OPLS all-atom model of n-decane [27]. The set of interaction parameters is collected in Table S1.

**Figure 6:**
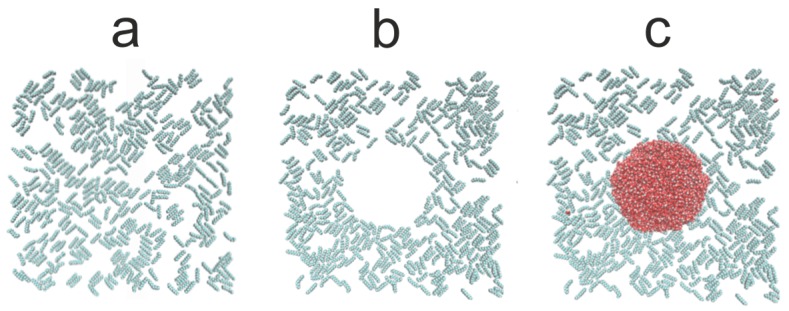
Simulation snapshots (top view) of water droplet on n-decane-covered silicon at C_HYDR_ = 0.73 molecules/nm^2^. The snapshot of the alkane on silicon before the water drop was introduced (**a**), the configurations of alkane molecules after the water drop was introduced ((**b**) water molecules are not shown), and the snapshots of water drop with the alkane molecules (**c**). Silicon surface is not shown for clarity.

**Figure 7:**
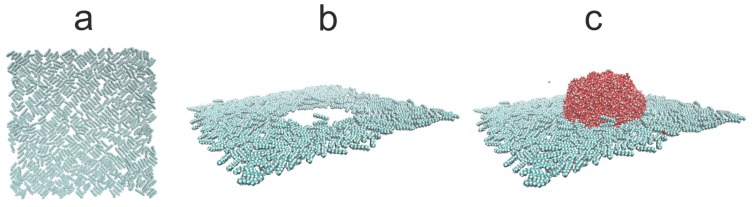
Simulation snapshots (side view) of water droplet on n-decane-covered silicon at C_HYDR_ = 1.29 molec/nm^2^. The snapshot of the alkane on silicon before the water drop was introduced (**a**), the configurations of alkane molecules after the water drop was introduced ((**b**) water molecules are not shown), and the snapshots of water drop with the alkane molecules (**c**). Silicon surface is not shown for better clarity.

**Figure 8:**
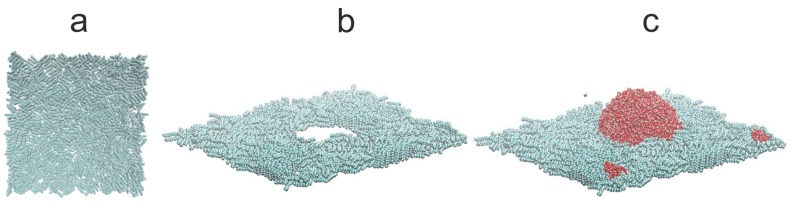
Simulation snapshots (side view) of water droplet on n-decane-covered silicon at C_HYDR_ = 2.92 molec/nm^2^. The snapshot of the alkane on silicon before the water drop was introduced (**a**), the configurations of alkane molecules after the water drop was introduced ((**b**) water molecules are not shown), and the snapshots of water drop with the alkane molecules (**c**). Silicon surface is not shown for better clarity.

We assume the additivity of diameters σ_ij_ = (σ_ii_+σ_jj_)/2 and the Lorentz–Berthelot mixing rule. The Coulomb and LJ interactions between atoms separated by three bonds within the same molecule were scaled down by multiplying them by 0.833333 and 0.5, respectively. All dispersion interactions have a cut-off radius r_cut_ = 1.5 nm, and the same distance was used to switch from a real-space to Fourier space calculations of the electrostatic interactions. The alkane–silicon system was allowed to equilibrate for 1.0 ns. Subsequently, 6000 SPC/E water molecules were placed above the alkane-covered silicon surface. The resulting water–alkane–silicon system was equilibrated for 10 ns followed by another 10 ns of gathering the time averages, and the same procedure of evaluating the WCA was adopted. Figure 6, Figure 7 and Figure 8 show simulation snapshots of an alkane covered silicon surface.

**Figure 9:**
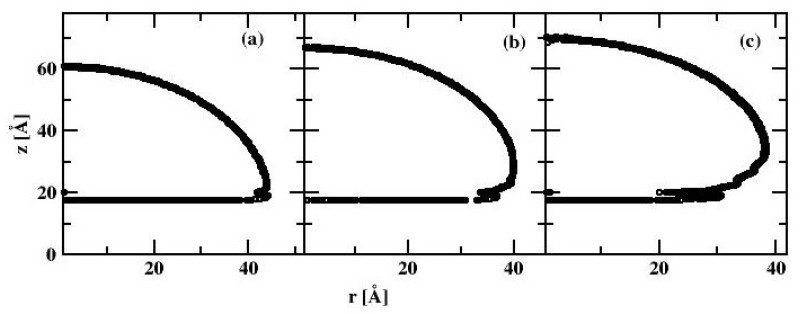
The spherical water droplet contour for the water-alkane-silicon system with 6000 molecules of water calculated at three hydrocarbon surface concentrations, C_HYDR_ = 0.73 (**a**), 1.29 (**b**), and 2.92 (**c**) molec/nm^2^. Circles denote the position of the water density profile with value 0.5 ± 0.03 g/cm^3^.

Without the water drop, the alkane molecules exhibit locally preferential orientation at 45 or 135 degrees wrt. the x-axis of the simulation box. This is particularly well visible at the C_HYDR_ = 1.29 molecules/nm^2^ (Figure 7). Once the water droplet is introduced it prefers to be in direct contact with the silicon surface, creating a hole in the alkane coverage. The so-called ”dimple” state [2] is preserved even at very high hydrocarbon surface coverage (cf. Figure 8). Figure 9 shows the water droplet contours calculated for three hydrocarbon surface concentrations, C_HYDR_ = 0.73, 1.29, and 2.92 molec/nm^2^.

All three profiles correspond to the ”dimple” state of the water nanodroplet. We observe that the WCA gradually increases with increasing C_HYDR_ with WCA = 88.7°, 103.6°, and 111.0°, respectively. The summary of simulations of water nanodrops on alkane covered bare silicon surface is presented in Figure 9, showing WCA dependence on the hydrocarbon surface coverage. We note that at low to moderate surface coverages WCA is practically independent of C_HYDR_. The WCA increase is observed at coverages close to a full monolayer (which is estimated to be 1.46 molec/nm^2^). Once the hydrocarbon surface density reaches 145% of monolayer coverage, i.e., 2.12 molec/nm^2^, WCA becomes independent of C_HYDR_ again with the elevated level of WCA around 111°.

### 3.4. Molecular Simulations of SPC/E Water on Silicon Covered with SiO_2_

**Figure 10:**
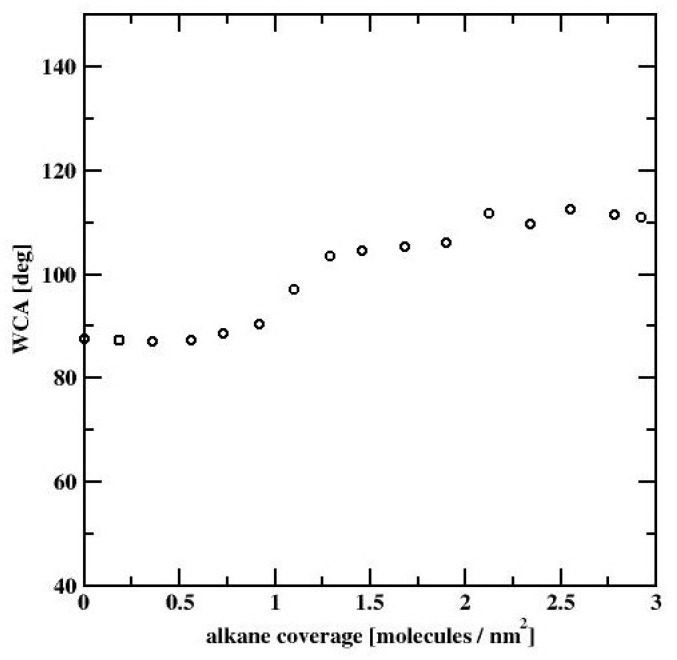
WCA dependence on hydrocarbon surface coverage, for water nanodroplets on n-decane-covered bare silicon surface.

The results shown in the previous section indicate an increase in the WCA with increasing hydrocarbon surface density (Figure 10). In order to recover the experimentally observed insensitivity of WCA to alkane surface coverage (Figure 2) a change in the surface–adsorbate interaction characteristics has to be introduced. We recall that the freshly etched silicon surface quickly develops a 1.4–1.5 nm thick film of silicon dioxide. This layer significantly alters the nature of the surface–adsorbate interactions by adding the electrostatic component to the dispersion interactions exerted by the bare silicon surface. In our modeling it is sufficient to consider only the SiO_2_ layer since we assume that the dispersion interactions have a cut-off at 1.5 nm and the underlying Si surface is located beyond the cut-off range. Our calculations are again divided into three stages. In the first stage, we determine the surface–adsorbate force field by considering cylindrical water nanodroplets and adjusting the interaction parameters, so that they match the experimental WCA values. In the second stage, we estimate how big the spherical nanodrop has to be in order to have negligible WCA finite size effects. Finally, in the third stage we determine the influence of the hydrocarbon surface density on water nanodroplets. In our calculations, we adopt the same computational procedures as those applied in the previous section.

**Figure 11:**
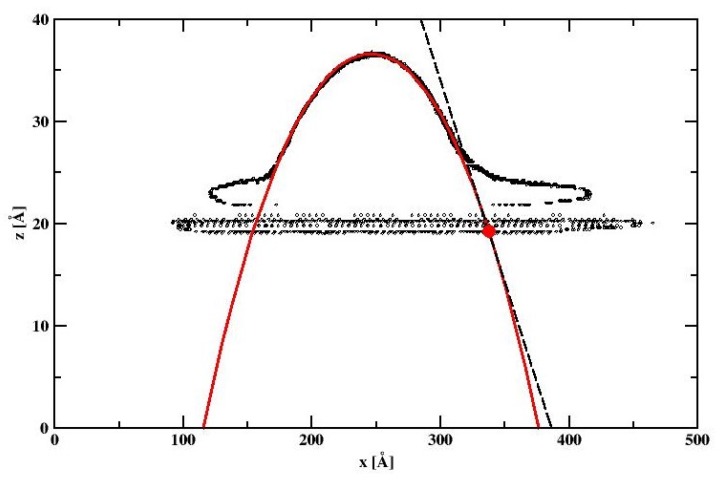
The cylindrical droplet contour calculated for the water–silicon dioxide surface system. The circles denote the position of the water density profile with value 0.5 ± 0.03 g/cm^3^. The thick red line denotes the droplet profile obtained by applying the fitting procedure. The dashed line denotes the line tangent to the drop that meets the bottom of the nanodroplet at the big red point. WCA dependence on hydrocarbon surface coverage for water nanodroplets on n-decane covered bare silicon surface.

Let us begin with cylindrical nanodrops on SiO_2_ surface. We assume that the surface atoms are completely immobile, whereas the interactions with water and hydrocarbon molecules have both the electrostatic and dispersion (LJ) components. In the literature, there are a number of different force fields for silicon dioxide [8,28,29,30,31]. For example, in [8] the charge associated with the silicon atom q_Si_ = 0.6e, whereas in [31], q_Si_ = 2.4e. In the present work, we started with the former value of the silicon charge and gradually increased it with accompanying decrease of the LJ energy parameter ε_Si_ so, that the experimental WCA was recovered. We have found that the set of the surface parameters q_Si_ = 1.1e ε_Si_ = 0.0502416 kJ/mole, σ_Si_ = 4.0534 Å, q_O,SiO2_ = −0.55e ε_O,SiO2_ = 0.286377 kJ/mole, and σ_O,SiO2_ = 2.8598 Å accurately reproduces the experimentally observed behavior of WCA, as will be shown below. The SiO_2_ substrate was composed by repeating the 12-atom α-crystoballite unit cell along x, y, and z-directions 108, 7, and 3 times, respectively. This process created a surface of the size 53.7624 nm × 3.4846 nm × 2.0844 nm. On the top of that surface 3000 SPC/E water molecules were placed and the simulation was carried out using the protocol described earlier. Figure 11 shows the contour of the cylindrical water nanodroplet on the SiO_2_ surface. 

**Figure 12:**
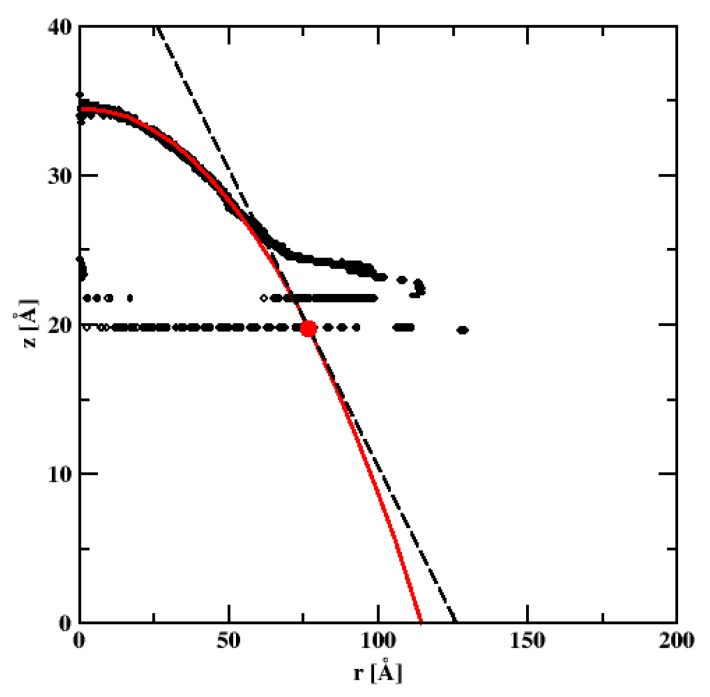
The spherical droplet contour calculated for the water–silicon dioxide surface system. The circles denote the position of the water density profile with value 0.5 ± 0.03 g/cm^3^. The thick red line denotes the droplet profile obtained by applying the fitting procedure. The dashed line denotes the line tangent to the drop that meets the bottom of the nanodroplet at the big red point.

**Figure 13:**
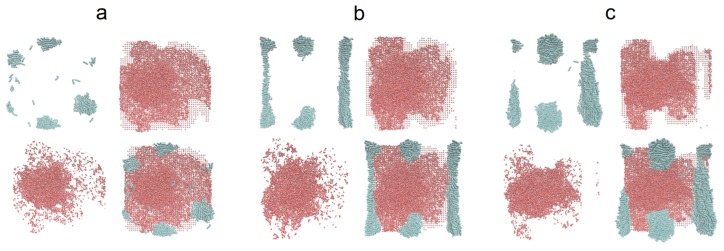
Simulation snapshots of water droplet on n-decane-covered silicon dioxide at surface density 0.59 (**a**), 1.47 (**b**), and 2.78 (**c**) molec/nm^2^. The top left-hand corner panel shows the snapshot of only the alkane molecules. The top right-hand corner panel shows only the water molecules. The bottom left-hand corner panel shows the water molecules but only those with z-coordinate greater than 2.55 nm. The bottom right-hand corner panel shows the snapshot of water nanodroplet with alkane molecules. The silicon dioxide surface is not shown for better clarity.

We note that there is a very thin (6Å thick) layer composed of water molecules very strongly adsorbed on the silicon dioxide surface. This is due to the strong electrostatic surface–adsorbate interactions. Nonetheless, a good profile of the nanodroplet can be obtained, which fits very nicely to the theoretical circular drop profile (cf. Figure 12, thick red line). From simulations we estimate WCA to be 21.6°. Figure 13 shows the contour of the spherical nanodroplet on the SiO_2_ surface evaluated for the surface comprising of 66,564 atoms obtained by replicating the α-crystoballite unit cell along x,y, and z directions 43, 43, and 3 times, and for 8000 SPCE water molecules. We note that, similar to the cylindrical nanodroplet, a thin layer of strongly adsorbed water molecules is easily detectable on the nanodroplet contour. Again, a well-defined nanodroplet fits very well to the theoretically expected circular drop profile (cf. Figure 12, red line). The WCA in this case equals 21.75° which is close to the WCA obtained for the cylindrical nanodroplet.

Finally, let us turn to water nanodroplets on alkane-preadsorbed SiO_2_ surfaces. In our calculations, we used 21.4054 nm × 21.4054 nm × 2.0844 nm surface comprising of 66,564 atoms obtained by repeating the α-crystoballite unit cell along x, y, and z directions 43, 43, and 3 times, and for 8000 SPCE water molecules. The simulation protocol was identical to that described in the previous section. Figure 13 shows the snapshots of water–alkane–silicon dioxide systems taken at three different hydrocarbon surface concentrations, C_HYDR_ = 0.59 (Figure 13a), 1.47 (Figure 13b), and 2.78 molec/nm^2^ (Figure 13c). At a first glance, one important difference to snapshots with the bare silicon surface (Figure 6, Figure 7 and Figure 8) is clearly noticeable. The n-decane molecules, instead of covering the whole surface, tend to group themselves into “islands” composed of stacked hydrocarbon chains (cf. Figure 13, top left-hand corner panels). The reason for such behavior is the strong electrostatic interaction between water molecules and the SiO_2_ surface. This leads to the formation of a thin layer of adsorbed water molecules covering the majority of the surface (cf. Figure 13, top right-hand corner panels). The alkane stacking minimizes the number of hydrocarbon–water contacts as they are unfavorable, and is furthermore promoted due to the weaker dispersion interactions between the n−decane and the surface. Water nanodroplets are located between the alkane “islands” (Figure 13, bottom left-hand corner panels).

**Figure 14:**
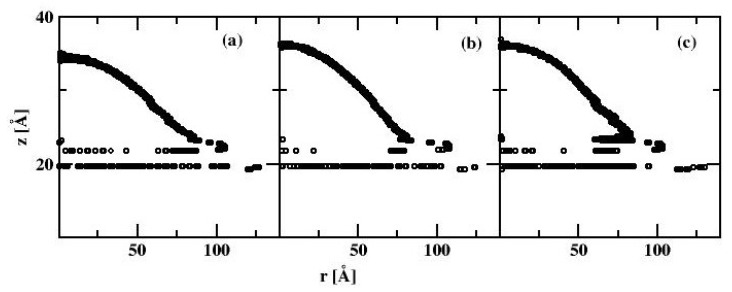
The spherical water droplet contour for the water–alkane–SiO_2_ system with 8000 molecules of water calculated at three hydrocarbon surface concentrations, CHY DR = 0.59 (**a**), 1.47 (**b**), and 2.78 (**c**) molec/nm^2^. The circles denote the position of the water density profile with value 0.5 ± 0.03 g/cm^3^.

Figure 14 shows the contours for the nanodroplets for systems presented in Figure 13a–c. Despite large differences in the hydrocarbon surface concentration, the nanodroplets are quite similar with WCAs equal to 18.8°, 21.8°, and 21.3° for C_HYDR_ = 0.59, 1.47, and 2.78 molecules/nm^2^, respectively.

## 4. Discussion

The major purpose of our study was to perform a holistic experimental and theoretical research on silicon wetting. Our XPS results (Figure 1) show that the SiO_2_ layer is present on our samples and the contents of other impurities can be neglected. This means that we expect hydrophilic nature of silicon covered by SiO_2_. The question arises if light airborne hydrocarbons adsorption occurs in remarkable amount. Hydrophilic nature of silicon surface is confirmed by the chromatographic experiment results (see Figure 3). Moreover, this experiment confirms that we expect remarkable influence of water on the repellence of light hydrocarbons from the droplet bottom. As it is proved, the method of sample purification has a strong influence on the WCA values (Figure 2). The most optimal method is the SDG procedure based on a series of treatments before a routine DG procedure. Obtained in this method WCA is equal to 20.7° and is the smallest value of our study. This WCA was next used as a reference for the calibration of the force field for the MD simulations. The differences in WCA values for the samples treated by using different methods are caused not by adsorption of light, but long hydrocarbons present in air. This is the reason why we observe only oscillations of WCA after exposure of silicon samples to C_10_H_22_ atmosphere. The simulation results show that the influence of hydrocarbons on the WCA of bare silicon should be remarkable, and the WCA increases by ~20° after exposure. In this mechanism, the repellence of hydrocarbons by water molecules from the bottom of the droplet at small coverages occurs. However, at larger surface hydrocarbons concentrations, water cannot repel hydrocarbons from the substrate and WCA increases. In this case the wetting mechanism is analogous to reported by us for a graphene [2]. In contrast for SiO_2_ covered surface, as light hydrocarbons are removed from the bottom of a droplet, the WCA is only slightly modified by hydrocarbons located at the droplet edge. In this case, hydrocarbons alter the shape of the contact line. Thus, the mechanism of wetting is similar, but not the same, to this observed recently for a strongly hydrophilic MOF [3]. Therefore, it can be stated that SiO_2_ covered silicon shows robust self-cleaning properties with respect to light hydrocarbons.

**Figure 15:**
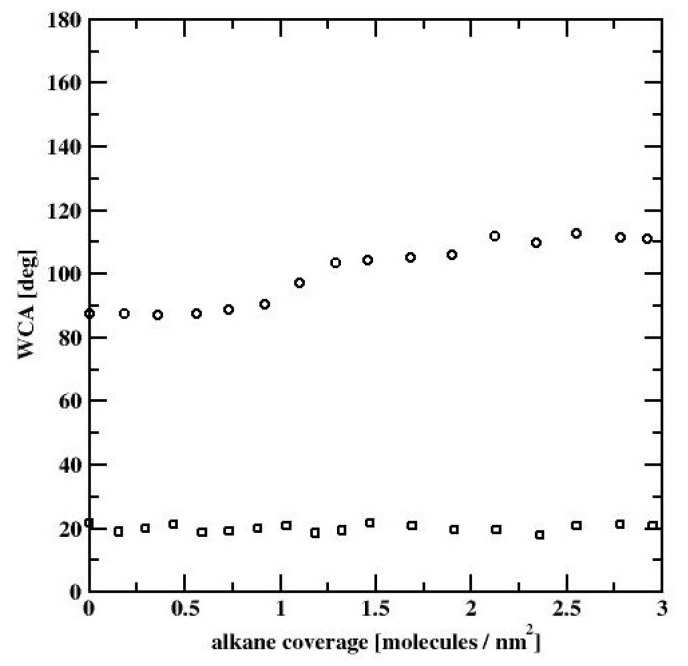
WCA dependence on hydrocarbon surface coverage, for water nanodroplets on n-decane covered bare silicon (circles) and silicon dioxide (squares) surfaces.

All our computational results are summarized in Figure 15, where we compare the WCA dependence on C_HYDR_ for the both, the bare silicon surface and the silicon dioxide surface. We note, that for the bare silicon surface (cf. Figure 15, circles), the WCA increases ~20° with the increase of the hydrocarbon surface density, whereas for the SiO_2_ surface (cf. Figure 15, squares), the WCA remains approximately independent of the alkane surface coverage, according to experimental data. The small (of about 3°) WCA variations in the latter case are due to the fact, that water nanodroplet is located between the alkane-rich domains. These alkane-rich domains may slightly alter the shape of the droplet leading to a small variation in the WCA. Thus, simulations provide molecular-level insight into the interaction between the airborne-adsorbed light hydrocarbons and water droplets. On freshly etched silicon surfaces, the strong dispersion interactions lead to strong adsorption of light hydrocarbons and ultimately to an increase of WCA. On the other hand, the thin layer of SiO_2_ present on all silicon surfaces when exposed to oxygen leads to significant screening of the dispersion component of the interactions. This promotes the electrostatic interactions between the thin layer of SiO_2_ and water molecules. Simultaneously this leads to a decrease in WCA and renders it almost insensitive to the airborne-adsorbed light hydrocarbons.

## 5. Conclusions

Our XPS study proves that the studied silicon surface is covered by a SiO_2_ layer. Among the studied methods of silicon surface purification, the SDG method is the most efficient. The WCA for (1,0,0) and (1,1,1) silicon surfaces are equal to 20.7 ± 2.7°. Light hydrocarbons strongly influence the wetting properties of bare, freshly etched silicon, and have small influence on the wetting of SiO_2_ covered surface. Molecular dynamics simulations reveal that the thin layer of SiO_2_ on the top of the silicon surface screens the dispersion interactions. This gives a rise to the weak adsorption of airborne light hydrocarbons and the insensitivity of WCA to the light hydrocarbon surface coverage.

The SiO_2_ covered silicon surface shows robust self-cleaning properties against light hydrocarbons. Therefore the differences reported in Figure 2 are caused by longer hydrocarbons remaining on a surface due to application of insufficient cleaning method. Based on our results those hydrocarbons are removed by modified de Gennes approach, according to the procedure described in this study.

## Figures and Tables

**Table 1:** The results of X-ray photoelectron spectroscopy (XPS) analysis.

Peak Name	Position (eV)	%At conc.	Species	
Si 2p 3/2 A	99.22	68.5		main silicon peak (Si 2p doublet)
Si 2p 1/2 B	99.82	-	Si (0)	second silicon peak (Si 2p doublet)
Si 2p B	103.2	24.7	Si (+IV)	SiO_2_
Si 2p C	101.63	0.8	Si (+III)	
Si 2p D	100.95	1.3	Si (+II)	
Si 2p E	100.41	1.3	Si (+I)	
Si 2p F	98.79	3.3	Si (0)	Deffective structures
O 1s A	532.6	94.7	O (-IV)	SiO_2_
O 1s B	531.4	5.3	O (-II)	hydroxides

## Supplementary Materials

The following are available online at https://www.mdpi.com/1996-1944/13/7/1554/s1, Figure S1: The cylindrical droplet contour calculated for the water–bare silicon surface system with kd = 0.147. The xc and zc. Cartesian coordinates denote the position of the water density profile with value 0.5 ± 0.03 g/cm^3^, Figure S2: Time dependence of the simulated water contact angle for the system with kd = 0.147. Table S1: Forcefield parameters applied during simulations of n-decane.
