# Supplementary material for: What Is the Value of Water Contact Angle on Silicon?
Source: Materials (Basel). 2020 Mar 27;13(7):1554. doi: 10.3390/ma13071554 (PMC7177545; doi:10.3390/ma13071554)
Supplement: Supplementary file 1 [file materials-13-01554-s001.pdf]

materials

MDPI

Article

What is the Value of Water Contact Angle on Silicon?

Paweł Bryk [1], Emil Korczeniewski [2], Grzegorz S. Szymański [2], Piotr Kowalczyk [3], Konrad Terpiłowski [4] and Artur P. Terzyk [2,*]

1 Department of Chemistry, Chair of Theoretical Chemistry, Maria Curie - Skłodowska University, 20–031 Lublin, Poland; pawel.bryk@gmail.com
2 Faculty of Chemistry, Physicochemistry of Carbon Materials Research Group, Nicolaus Copernicus University in Toruń, Gagarin Street 7, 87–100 Toruń, Poland; e.korczeniewski@umk.pl (E.K.); greg_ss@umk.pl (G.S.S.); aterzyk@chem.umk.pl (A.R.T.)
3 College of Science, Health, Engineering and Education, Murdoch University, WA 6150, Australia; kowalczyk.piotr@wp.pl
4 Department of Chemistry, Chair of Physical Chemistry of Interfacial Phenomena, Maria Curie - Skłodowska University, 20–031 Lublin, Poland; terpil@poczta.umcs.lublin.pl
* Correspondence: aterzyk@chem.umk.pl; Tel.: +48–56–61–14–371

Received: 4 March 2020; Accepted: 26 March 2020; Published: date

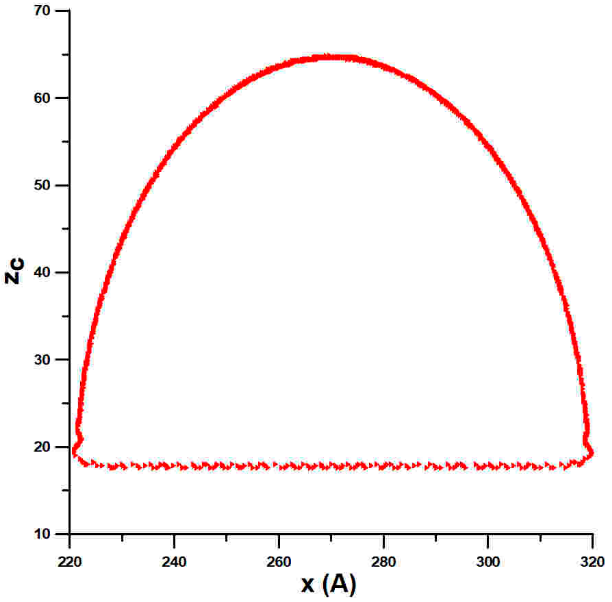

Figure S1. The cylindrical droplet contour calculated for the water- bare silicon surface system with k_d = 0.147. The x_c and z_c. Cartesian coordinates denote the position of the water density profile with value 0.5 ± 0.03g/cm^3.

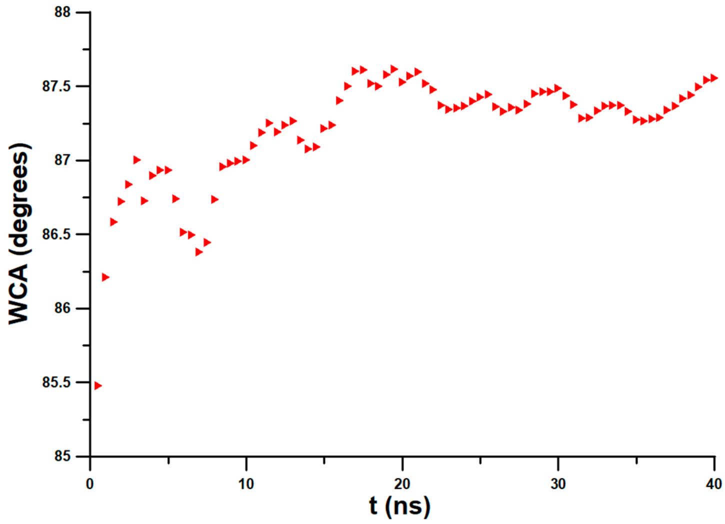

Figure S2. Time dependence of the simulated water contact angle for the system with k_d = 0.147.

Table S1. Forcefield parameters applied during simulations of n-decane.

Parameter	Value
σ_C	3.5 Å
ε_C	0.276144 kJ/mole
q_C^{CH2}	-0.12e
q_C^{CH3}	-0.18e
q_H	0.06e
ε_H	0.125520 kJ/mole
l_{CC}	0.1535 nm
k_{CC}	224262.4 kJ/(mole nm^2)
l_{CH}	0.10930 nm
k_{CH}	284512.0 kJ/(mole nm^2)
Θ_{HCH}	1.88146493 rad
Θ_{HCC}	1.93207984 rad
Θ_{CCC}	1.96698607 rad
k_{HCH}	276.144 kJ/(mole rad^2)
k_{HCC}	313.800 kJ/(mole rad^2)
k_{CCC}	488.273 kJ/(mole rad^2)
$c_{HCCC}^{(0)}$	0.6276 kJ/mole
$c_{HCCC}^{(1)}$	1.882800 kJ/mole
$c_{HCCC}^{(2)}$	0.0000 kJ/mole
$c_{HCCC}^{(3)}$	-2.510400 kJ/mole
$c_{HCCH}^{(0)}$	0.6276 kJ/mole
$c_{HCCH}^{(1)}$	1.882800 kJ/mole
$c_{HCCH}^{(2)}$	0.0000 kJ/mole
$c_{HCCH}^{(3)}$	-2.510400 kJ/mole
$c_{CCCC}^{(0)}$	2.9288 kJ/mole
$c_{CCCC}^{(1)}$	-1.4644 kJ/mole
$c_{CCCC}^{(2)}$	0.2092 kJ/mole
$c_{CCCC}^{(3)}$	-1.6736 kJ/mole
